# The GARD Prebiotic Reproduction Model Described in Order and Complexity

**DOI:** 10.3390/life14030288

**Published:** 2024-02-21

**Authors:** Christian Mayer, Doron Lancet, Omer Markovitch

**Affiliations:** 1 Institute of Physical Chemistry, CENIDE, University of Duisburg-Essen, 45141 Essen, Germany; 2 Department of Molecular Genetics, The Weizmann Institute of Science, Rehovot 7610001, Israel; doron.lancet@weizmann.ac.il; 3 Blue Marble Space Institute of Science, 600 1st Avenue, Seattle, WA 98104, USA; omermar@gmail.com

**Keywords:** GARD, mutually catalytic networks, attractor dynamics, evolution, order, complexity, origin of life, molecular evolution, prebiotic chemistry, statistical thermodynamics, entropy, population dynamics

## Abstract

Early steps in the origin of life were necessarily connected to the unlikely formation of self-reproducing structures from chaotic chemistry. Simulations of chemical kinetics based on the graded autocatalysis replication domain (GARD) model demonstrate the ability of a micellar system to become self-reproducing units away from equilibrium. Even though they may be very rare in the initial state of the system, the property of their endogenous mutually catalytic networks being dynamic attractors greatly enhanced reproduction propensity, revealing their potential for selection and Darwinian evolution processes. In parallel, order and complexity have been shown to be crucial parameters in successful evolution. Here, we probe these parameters in the dynamics of GARD-governed entities in an attempt to identify characteristic mechanisms of their development in non-covalent molecular assemblies. Using a virtual random walk perspective, a value for consecutive order is defined based on statistical thermodynamics. The complexity, on the other hand, is determined by the size of a minimal algorithm fully describing the statistical properties of the random walk. By referring to a previously published diagonal line in an order/complexity diagram that represents the progression of evolution, it is shown that the GARD model has the potential to advance in this direction. These results can serve as a solid foundation for identifying general criteria for future analyses of evolving systems.

## 1. Defining Progress in GARD Evolution

Initial steps in the origin of life include the formation of ordered and complex structures from more or less chaotic chemistry [1,2,3,4,5,6,7,8,9,10]. Such a development requires a special driving force, either represented by a random formation and selection process or connected to an autocatalytic network that favors the formation of so-called attractors, exemplified by a specific type of living cell that preserves a recurrent pattern [11]. Motivated by the latter and based on the likely presence of various amphiphilic compounds in a prebiotic environment, the graded autocatalysis replication domain (GARD) model has been proposed [12,13,14,15,16,17]. Within the GARD model, attractor dynamics were shown to drive towards micellar self-reproducing states with very specific amphiphile compositions, so-called composomes [15]. Similar to RNA strands, composomes have the capability to carry and multiply coded information [14]. Instead of being represented by a molecular chain sequence as in RNA, composomes code compositional information [12] in the specific deviation of the relative amounts of the various amphiphiles in the micelle from those in the surrounding solution. These characteristic deviations from the surrounding solution, caused by mutual catalysis among molecules in a micelle formation, are thus a direct consequence of the specific catalytic network [12,13,14,15,16,17]. Different composomes are different attractor states of the internal organization of such networks [17]. In other words, micelle replication in GARD is driven by self-assembly and biased due to the micelle composition and catalytic network (somewhat resembling supramolecular complexification). Finally, while the GARD model was intuitively developed with the application of lipids in mind, hence the term “Lipid World”, it was recently suggested that a system of supramolecular fibers in which self-replication is driven by self-assembly is analogous to the GARD model [18].

**Figure 1:**
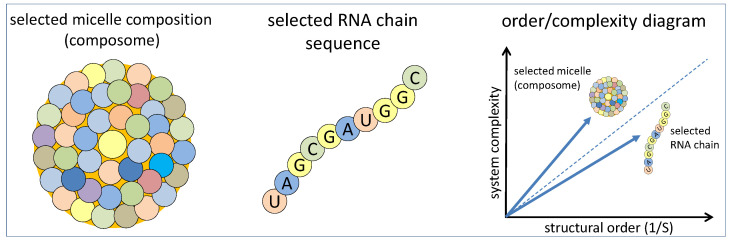
A composome made up of 20 types of amphiphilic molecules at different amounts (**left**). The relative amounts are significantly different from the surrounding solution and can code as much information (20 bit) as a sequence of ten bases in a selected RNA chain (**center**). The formation of either structure would represent significant progress in an order/complexity diagram, where the diagonal line stands for evolution towards functional systems (**right**).

In the simplest case, the information is limited to relative increases and decreases in amphiphile concentrations within the accreting micelle compared to the environment. If we simply assign the value “1” to an increased (or unchanged) concentration and a value “0” for a decreased one, the information coded in a single composome consists of as many bits as the count of amphiphile types. In a given example, a composome made up of twenty different types of amphiphiles that have been either enriched (“1”) or depleted (“0”) with respect to the concentrations in solution carries the information of 20 bits (Figure 1 left), which corresponds to an RNA strand with 10 bases (Figure 1 center). In the case of the composome, the amount of information increases with the possibility of introducing various degrees of change in amphiphile relative concentrations. As the term “composome” suggests, this coded information may be considered a primitive version of a genome of a given species. 

Lately, the two parameters, order and complexity, have been proposed as principal criteria to describe the course and the progress of evolution [19]. The idea behind that approach is that order without complexity leads to a non-functional crystalline state, whereas complexity without order leads to non-functional molecular chaos. Only a combination of order and complexity can achieve a certain degree of functionality and represent a significant step towards life. 

We stress that the word “composome” means *self-reproducing* compositional assembly, whereby only very few compositional assemblies are composomes. This is, in fact, true also for RNAs: only very few (so far none observed) can self-replicate. As for evolutionary progression with a concomitant increase in order and complexity, lengthening a single molecule of replicating RNA will not serve to approach life, while the reproducing micelles (with just adding catalyzed covalent reactions) can do it very well in this respect, leading to progress toward metabolic GARD [12].

From this perspective, both systems, self-reproducing composomes and self-replicating RNA chains, have the potential to evolve towards functionality (which per se needs to be ordered and complex). In an order/complexity diagram (Figure 1 right), such a development would ideally follow a diagonal line with a positive slope [19]. The concept of an RNA world suggests that RNA strands possess this potential through a process of molecular evolution [20]. In the following, we demonstrate that the GARD model based on the action of a mutual catalytic network opens this perspective as well, even though the characteristics of the development may differ significantly. In any case, order combined with complexity is the main criterion that decides if and how efficiently the GARD process can lead to a successful evolution. Moreover, predictions on possible long-term developments toward functional systems can be made through this perspective. 

## 2. Representation of the GARD Model: A Basic Rate Equation

All developments within the GARD model are determined by the principles of mass-action chemical kinetics. The formation of a micelle assembly with mutually-catalytic reproduction follows a set of differential equations [12]:

(1) $$\frac{dn_{i}}{dt}=\left(k_{i}\pi_{i}N-k_{-i}n_{i}\right)\left(1+\frac{1}{N}\sum_{j=1}^{N_{G}}\beta_{ij}n_{j}\right)$$

with *N_G_* being the number of amphiphilic molecule types indexed *i*, which occur at an external concentration π*_i_*, *n_i_* the count of molecules of type *i* in the micelle, *N* being the total number of molecules in the micelle, *k_i_* and *k_−i_* the corresponding forward and backward rate constants for the integration into the assembly, and the matrix elements *β_ij_* describing the matrix of specific rate enhancement parameters caused by mutual catalysis between molecule types *i* and *j*. 

At this point, the actual challenge is to analyze the GARD process in the context of the order and complexity parameters, as performed before for the RNA world [20]. As a first step, it is necessary to define such parameters for the GARD assembly process, as given by a time development according to Equation (1).

## 3. Defining the Order of the System—Reciprocal Sequential Entropy

For the description of the GARD model in terms of order, we stick to a similar approach as in the case of the RNA world [20]. In fact, the main difference between both cases is the fact that the sequential structure of the RNA is replaced by the compositional structure of the GARD assemblies. As a manifestation of the system’s order, we will again use the reciprocal entropy assigned to the entity of all random walks through the whole system, which, in these cases, consists of aqueous amphiphiles as well as amphiphiles self-organized in micelles. We should emphasize that these random walks are merely a tool for the evaluation of order and complexity; they have nothing to do with the actual accretion process in the system.

Each random walk follows a given set of rules:

(1) The starting point may be any amphiphilic molecule of the system.
(2) In each step, the move preferably occurs to the closest adjacent unit (nearest neighbor), which may be either in solution or part of a micelle.
(3) If rule two leads to a unit that is already part of the given random walk, choose the one with the next shortest distance. This rule applies repeatedly until the next step leads to a “fresh” unit that has not been part of the random walk so far.
(4) The random walk stops after *N_rw_* steps, with *N_rw_* being the total number of units in the system.
(5) The random walk is repeated multiple times within a specific time frame to account for diffusion processes in the system.

These rules guarantee that the resulting set of pathways reflects a characteristic cross-section of the system. Rule no. one allows for the definition of different pathways until the full system is specified. Rule no. two accounts for contacts and nearest neighborhoods resulting from local interactions as well as brief contacts between molecules. Rule no. three efficiently prevents the formation of closed loops. Of course, the random walks will vary over time as the system undergoes dynamic changes. The time window of the random walks according to rule no. five is chosen such that molecular diffusion is possible while no micellar compositions are changed. Under these conditions, the full set of pathways will represent a complete image of the system’s micellar structure. It will account for preferred intermolecular contacts, surface interactions, activated complexes, etc. There may be alternative concepts allowing for structural monitoring of the system, but the described procedure should be among the most efficient strategies. It creates a virtual string of amphiphiles that forms a reliable basis for quantitative analysis of the system’s order and complexity.

Initially, we focus on a determination of the degree of order. Following the rules of statistical thermodynamics, we derive a general expression for this characteristic “sequential” entropy encountered in such a set of random walks based on a set of system parameters:

(1) The number *N_G_* of different types of amphiphiles.
(2) The relative contributions (fractional concentration) *π_i_* of all given amphiphile type *i* in solution (with *π*_1_ + *π*_2_ + *π*_3_ + … + *π_NG_* = 1). In the case of equal concentrations of all amphiphiles, we obtain *π*_1_ = *π*_2_ = *π*_3_ = … = *π_NG_* = 1/*N_G_*.
(3) The average number of aqueous amphiphilic molecules *M* between two micelles in the random walk (*M* may decrease over time with increasing micelle concentration).
(4) The average total number of amphiphilic molecules in a micelle *N*.
(5) The predictability *p_i,k_* of a given type of amphiphilic molecule *i* in accordance with the composition of the micelle population *k*. In general, *p_i,k_* is identical to the relative contribution of the amphiphile *i* in the micelle population *k* (which in the GARD model could correspond to an individual composome within the ensemble).
(6) The average total number *N_k_* of amphiphilic molecules in a micelle belonging to the population *k*.
(7) The relative contribution *P_k_* of micelle population *k* with respect to all micelles.
(8) The total number *N_rw_* of units in the system and on the pathway of the random walk.

With these parameters given, the characteristic sequential entropy contribution *S_r_* of a random walk is derived as (for a justification of Equation (2), see Supplementary Materials):

(2) $$S_{r}=k_{B}\;\ln\;w=k_{B}\;M\left(\frac{N_{rw}}{M+N}\right)\sum_{i=1}^{N_{G}}\pi_{i}\ln(1/\pi_{i})+\;k_{B}\;\sum_{k}\left[\left(\frac{P_{k}N_{rw}}{M+N}\right)N_{k}\sum_{i=1}^{N_{G}}p_{i,k}\ln\left(1/p_{i,k}\right)\right]$$

with k_B_ as Boltzmann’s constant and *w* as the statistical weight defined by the variety within the set of random walks. If *N_rw_* is divided by Avogadro’s number (N_A_ ≈ 6.022 × 10^23^), *S_r_* is transformed into a molar quantity (with respect to the amphiphilic molecules) and can be given in units of J/(K·mol). The reciprocal value 1/*S_r_* (in K·mol/J) finally characterizes the order of the system. 

In the case of a constant monomer pool and a constant micelle concentration (*M* = const.) as well as equal and time-independent contributions *π_i_* of all amphiphiles *i*, the first part of Equation (2) becomes constant and may initially be disregarded as a contribution to the time development of *S_r_*. The actual time dependence of *S_r_* is then determined by the (possibly growing) deviation of the individual micellar composition *p_i,k_* from the original equilibrium composition *π_i_*. In a GARD development, the formation of a composome may possibly lead to an initial increase in *S_r_*, corresponding to a decrease in order, especially with unequal starting concentrations *π_i_*. However, with increasing reproduction of this composome and correspondingly increasing pre-factor *P_k_* for its more or less defined composition, the order will rise significantly. When compared to the situation for selected RNA chains [20], it is the distinct *composition* of the composome that now plays the role of coded information, similar to the chain *sequence* in RNA.

In principle, Equation (2) can be applied to every single micelle in the system such that the index *k* would refer to individual micelles (and *N_k_* = 1 for all *k*), an approach that is preferentially used on simulated GARD processes (see Section 5.3). In the case of experimental data, it makes more sense to identify groups of micelles (composomes) and address them using the index *k*, accepting a certain variation in their composition *p_i,k_*. We call such groups “compotypes”, as defined [12]. 

Crucially, the GARD dynamic behavior always stays away from equilibrium. This is due to the realistic assumption that the environment is buffered, meaning that there is always a supply of amphiphiles above the critical micellar concentration (or, by the same token, critical vesicle concentration). The accretion reaction that makes micelles grow is definitely downhill, i.e., with negative free energy change, stemming from the hydrophobic interactions of tail–tail adherence in water. At later evolutionary stages, as detailed below, the supply of high-energy compounds keeps the non-equilibrium for endogenous covalent syntheses. Finally, what always keeps the micellar system away from equilibrium is the fission process, driven by external forces such as turbulence, allowing restarting the energetically downhill progression of accretion [12,13,14,15,16,17].

## 4. Defining the Complexity of the System—The Size of the Reproducing Algorithm

Among the many approaches to determining system complexity as a parameter, the idea originally developed by Andrey Nikolaevich Kolmogorov seems to be most appropriate to characterize prebiotic development [21,22,23,24]. It relies on the assumption that, for every structure, there is a minimal size of an algorithm (or computer program) that fully describes its entity and all of its details. The size of this computer algorithm in a universal description language in bits or bytes may then serve as a measure for the degree of complexity of the system. Although it is difficult or even impossible to determine the minimal size of the algorithm exactly (a problem known as Chaitin’s incompleteness theorem [25]), this number can still be approximated for a molecular system [19,20]. The challenge in the given case is to describe a model algorithm that fully reproduces the statistics of the sequences within a corresponding set of random walks. Its Kolmogorov complexity is then determined by its minimum size in bits or bytes [21,22,23,24]. 

The code needed to define the procession of the aqueous amphiphiles could involve the repeated action of a simple random number generator with an output of (statistically weighted) integer numbers between one and *N_G_*. Hence, it is limited to a few bits and can be neglected under the given circumstances. Initially, the same holds for micelles that form randomly by amphiphilic interaction. Their composition is represented by the given set of starting concentrations *π_i_*. Under these conditions, the random walk through the micelles follows the same rules as the one through the aqueous amphiphiles, such that the reproduction of the overall data string would not require any additional code. 

This changes dramatically as soon as the catalytic network starts to influence the micellar composition, according to Equation (1). During this development, the micellar composition is no longer random but follows the principle of an attractor [15], leading to amphiphile populations that deviate significantly from the starting values *π_i_*. The resulting composome attractors take an increasing influence on the data string of the random walk. In a reproduction of the string statistics, their presence has to be accounted for; therefore, they represent a corresponding contribution to the system’s complexity. In the following, we propose an analytical approach to estimate the additional complexity associated with the formation of composomes. 

Numerical simulations of micellar attractors have shown that composomes that emerge from an environment with *N_G_* lipid types generally end up with the specific participation of a characteristic subset of types *N_C_* < *N_G_* [15,26]. All other amphiphiles that occur at the equilibrium concentration do not require additional code for the individual composome’s description. This given, the full compositional variability of a composome indexed *k* characterized by a subset *N_Ck_* of different types of amphiphiles is determined by the combinatorial expression [12]:

(3) $$\left(\begin{array}{c} N_{Ck}+N_{k}-1\\ N_{k} \end{array}\right)=\frac{\left(N_{Ck}+N_{k}-1\right)!}{N_{k}!\left(N_{Ck}-1\right)!}$$

which would lead to a corresponding contribution *c_k_* to the Kolmogorov complexity (and a total system complexity *c*) over a random walk:

(4) $$c_{k}=P_{k}\left(\frac{N_{rw}}{M+N}\right)\;\log_{2}\;\frac{\left(N_{Ck}+N_{k}-1\right)!}{N_{k}!\left(N_{Ck}-1\right)!}\;\mathrm{and}\;c=\sum_{k}c_{k}\left(\mathrm{in}\;\mathrm{bit}\right)$$

If *N_rw_* is divided by Avogadro’s number (N_A_ ≈ 6.022 × 10^23^), *c* is transformed into a molar quantity (with respect to the amphiphilic molecules) and can be given in units of bit/mol (or byte/mol if divided by 8). 

Basically, that means that an individual digital code is assigned to each possible attractor composition and that the number of such digital codes needed for each composome depends on the population *P_k_* as well as on the number and the variety *N_k_* and *N_Ck_* of amphiphiles of the specific composome *k* in the system. When applied to a simulated GARD development (see Section 5.3), an individual index *k* is assigned to every single micelle while the corresponding *c_k_* is equal to the amount of code necessary for the description of its composition. The overall expectation for the GARD development is a step-by-step increase in complexity over time, each step representing the formation of a new type of composome. The height of each step depends on the new composome’s characteristics: generally, it will be smaller for composomes with a narrow compositional distribution, which, on the other hand, will lead to a larger increase in the order in the system (and vice versa). 

## 5. Application to GARD Developments

A clean analysis of the GARD development with respect to order and complexity requires a model calculation for the full-time development of a micellar ensemble according to the differential Equation (1) (see Section 5.3). Initially, based on the considerations mentioned above, one may roughly sketch a few typical steps that are expected to occur during the process. 

### 5.1. Composome Formation

**Figure 2:**
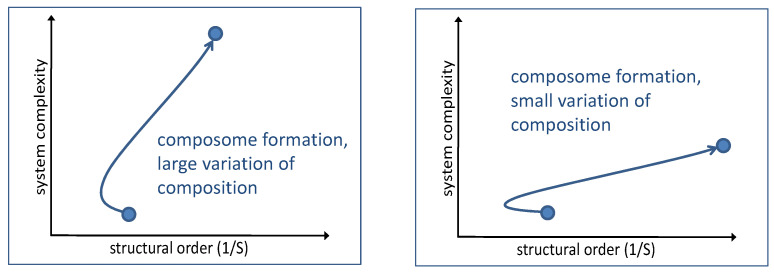
Expected qualitative order–complexity diagrams describing an initial composome formation for wide (**left**) and narrow (**right**) compositional variation.

As the natural first step, we consider the initial formation of a single composome (Figure 2). The starting condition is represented by a random seeded assembly with the set of amphiphile fractional concentrations approximately matching those in the environment, *π_i_*. Correspondingly, the order of this state is determined according to Equation (2), being lowest for the balanced distribution *π*_1_ = *π*_2_ = *π*_3_ = … = *π_NG_* = 1/*N_G_*. The system complexity is quite low as well: the string of amphiphiles along the course of the random walk can be reproduced by the repeated action of a simple random number generator. Accordingly, the Kolmogorov complexity is probably in the range of a double-digit number in bits. 

In any case, the formation of a composome will initially be connected to a certain loss in order, at least in the case of uneven amphiphile partitions, *π_i_*. The action of the catalytic network (as determined by the matrix β) leads to reproduction; hence, an additional fraction of micelles represent the first composomes (*k* = 1), with a corresponding distribution function *p_i,_*_1_ that needs to enter Equation (2). In general, *p_i_*_,1_ will differ from *π_i_*, which necessarily leads to a wider variability of next neighbors in a random walk, hence, to a lower order. At the same time, the complexity will increase as the characteristic composition of the composome will ask for additional code, as described by Equation (4). With increasing action of the dynamic attractor, the composome will be approached, leading to a large *P_k_* for this variety in Equation (4). In addition, reproducing composome attractors tend to have strongly polarized compositions (narrower repertoire of molecule types appearing in the micelle), which further decrease their contribution to the sequential entropy, thus leading to a higher order. With a wide variety of compositions, the gain in order will be small, but the increase in complexity will be large. With a narrower composition, i.e., a smaller number of different molecule types, the system will gain more order but make a smaller step in complexity. In an order/complexity diagram, both developments will follow the course of a successful evolution, with some dependence on the given compositional characteristics (Figure 2). 

### 5.2. Formation of Additional Composomes

**Figure 3:**
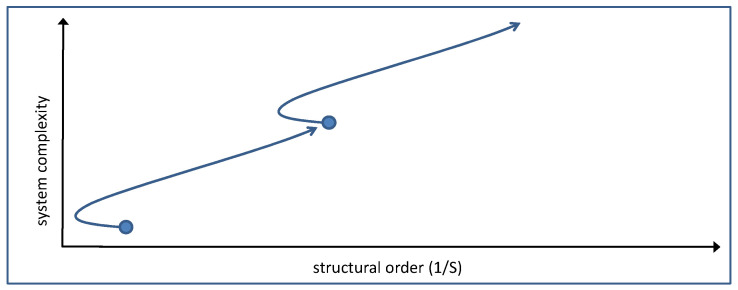
Expected qualitative order–complexity diagram for subsequent composome formations.

A micellar system may reach the reproducing composome dynamic state as determined by the inner structure of the *β*-matrix [17]. As the system evolves, additional types of composomes may form subsequently. Each such step would again follow the same typical pattern (Figure 3) as more environmental individual molecules accrete to orderly composomes, which are more complex than randomly scattered individual molecules. Initially, the complexity of the system would increase, and its order would decrease due to the appearance of an additional attractor composition. In the following, the new composome would be replicated by growth–split dynamics, leading to a significant increase in both parameters. The order–complexity plot again follows the pattern of successful evolution, resulting in a higher state of functionality. 

### 5.3. Application to Simulated GARD Developments: Composome Takeover

**Figure 4:**
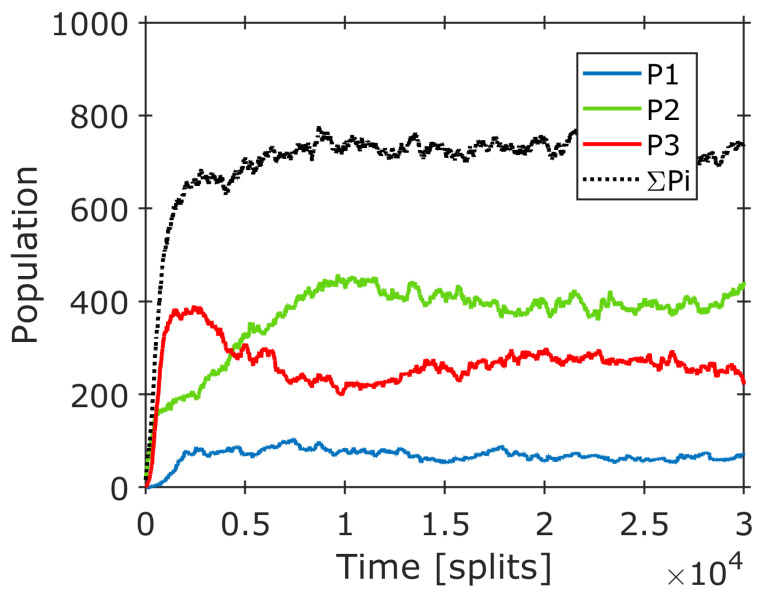
Simulated data for simultaneous composome formation and a relatively slow and mild takeover. The colored plots show the propensities *P_k_* of composomes *k* within a constant population reactor for three composomes (*P*_3_—red; *P*_1_—blue; *P*_2_—green). The black dotted line marks the sum of all composome contributions (*P*_1_ + *P*_2_ + *P*_3_). A slow takeover is observed between red and green, the latter overcoming the former and becoming dominant at steady state [26].

In the following, the order/complexity concept is applied to computer simulations of GARD developments based on Equation (1). Calculated assemblies are generally more complicated as, depending on the β-matrix, several composomes tend to proliferate simultaneously. Quite often, a composome type A that rapidly forms at the beginning is taken over by a composome type B that dominates in the final steady state. The term “takeover” in this sense stands for a process where the increase in population B is accompanied by a decrease in population A; hence, amphiphiles are being shifted from A to B. Realistic examples of simultaneous composome formations together with composome takeovers were presented in previous studies [12,26]. An example of a relatively slow and mild takeover is shown in Figure 4. 

A numeric simulation over the period of 30,000 growth–split events shows the initial formation of composome 3 (Figure 4, red), followed by the subsequent formation of composomes 1 (Figure 4, blue) and two (Figure 4, green). The takeover occurs continuously between 2500 and 10,000 time steps. During the whole process, a continuous increase in the overall composome population is observed (dotted line in Figure 4). 

**Figure 5:**
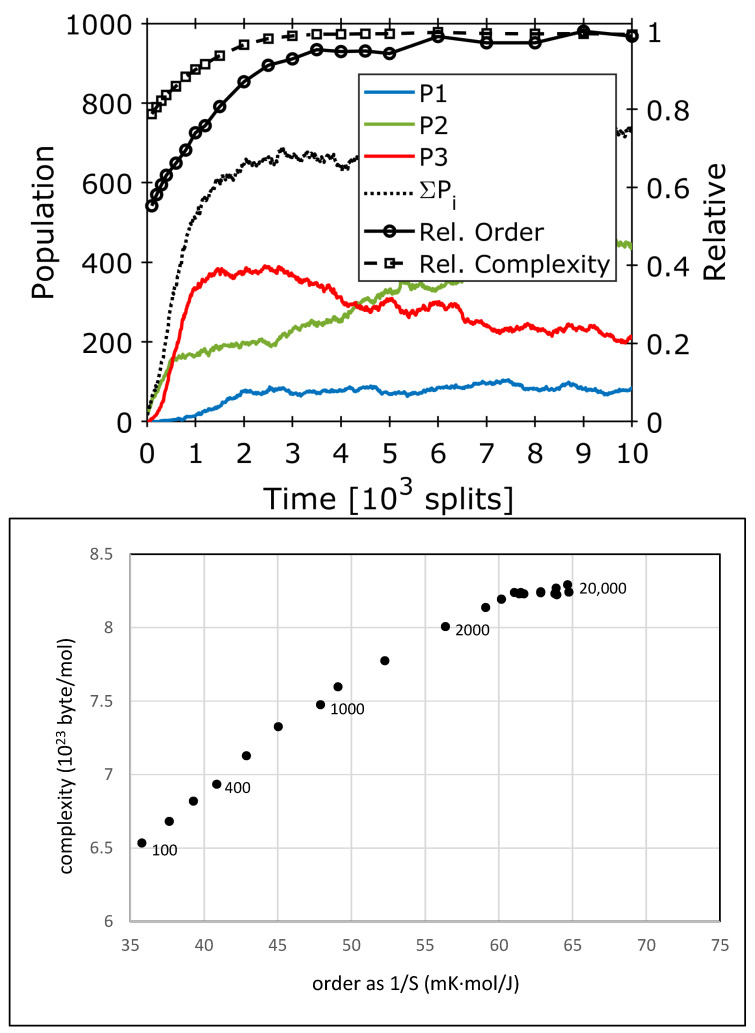
(**Top**): Initial part of the simulated composome development for a slow and mild takeover as shown in Figure 4, together with calculated values for order and complexity according to Equations (2) and (4). Order and complexity are plotted as relative values with respect to their corresponding maximum. (**Bottom**) Resulting order–complexity diagram for 20,000 time steps (in absolute values for one mole of amphiphile). Numbers next to data points indicate the number of time steps, as shown in Figure 4. Due to the superposition and the mild characteristic of the takeover, the development of the three composomes is not resolved but leads to a continuous increase in both values, leveling out above 10,000 time steps.

The consequences of this development on the order and complexity of the system are calculated according to Equations (2) and (4) and are introduced as relative values in Figure 5 (top). An initial loss of order (as shown in Figure 2 and Figure 3) is missing as the simulation starts with an even distribution over all amphiphiles. During the time interval 0 < t < 2500, all composomes form simultaneously, leading to a corresponding increase in order and complexity. After 3000 time steps, composome 2 is continuously replacing composome 3; therefore, order and complexity remain more or less constant. In the order/complexity diagram (Figure 5, bottom), a continuous increase in both parameters is observed up to 10,000 time steps. In a superposition, the consecutive formation of the three composomes is not resolved in the order/complexity plot; instead, it appears as a single development. Above 10,000 time steps, both parameters level out to a constant value. Overall, the complexity increases by 30%, while the order almost doubles (+80%). In absolute values, the gain of order corresponds to a negative entropy change of −12.48 J/(K∙mol), referring to the amount of amphiphile. All in all, the observed procedure is a clear step towards increased order and complexity and, hence, a valid step in an evolution process.

**Figure 6:**
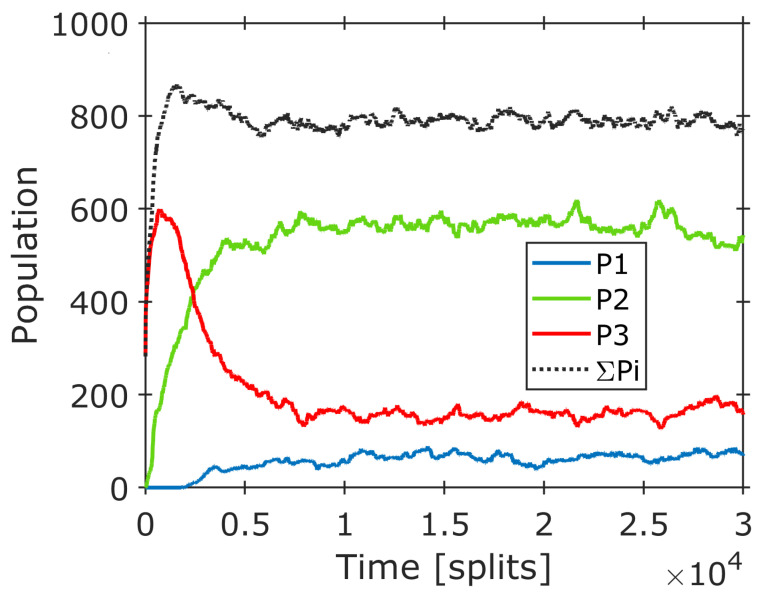
Simulated data for simultaneous composome formation with a rapid and dramatic takeover. The colored plots show the propensities *P_k_* of composomes *k* within a constant population reactor, for three composomes (*P*_3_—red; *P*_1_—blue; *P*_2_—green). The black dotted line marks the sum of all composome contributions (*P*_1_ + *P*_2_ + *P*_3_). A dramatic takeover is observed between red and green, the latter overcoming the former and becoming dominant at steady state [26].

Other settings of the β-matrix lead to compositional changes that are more dramatic. Figure 6 shows an example of a rapid takeover between composomes. In this case, a numeric simulation of over 30,000 growth–split events reveals the initial formation of composome 3 (Figure 6, red), followed by the subsequent formation of composomes 1 (Figure 6, blue) and two (Figure 6, green). Below t = 1000, composome 3 dominates the micellar composition. Within only 5000 time steps, composome 3 is rapidly taken over by composome 2. The sum of all populations (black dotted line in Figure 6) rapidly increases and goes through a maximum at1500 time steps. 

**Figure 7:**
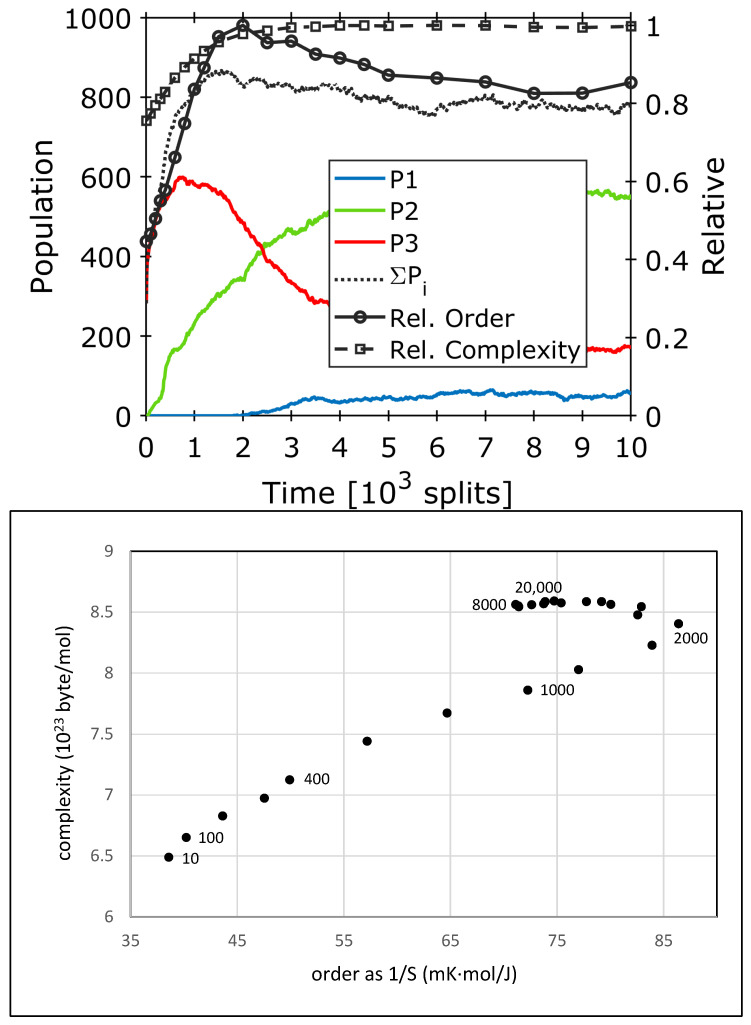
(**Top**): Initial part of the simulated composome development for a rapid and more dramatic takeover as shown in Figure 6, together with calculated values for order and complexity according to Equations (2) and (4). Order and complexity are plotted as relative values with respect to their corresponding maximum. (**Bottom**): Resulting order–complexity diagram for 20,000 time steps (in absolute values for one mole of amphiphile). Numbers next to data points indicate the number of time steps, as shown in Figure 6. The more dramatic takeover process near t = 2000 leads to a significant loss of order up to t = 8000, while complexity still rises. The system order only partially recovers when the data level out at t = 20,000.

The evaluation of the rapid takeover process regarding order and complexity is depicted in Figure 7. Up to 2000 time steps, the takeover process is characterized by a continuous increase in order and complexity. This is mainly caused by the steep increase in the population of composome 1 as well as the formation of the new composome 2, which overcompensates the loss of composome 3. Order reaches a maximum at 2000 time steps. For 2000 < t < 8000, the significant loss of composome 3 causes a partial inversion of the increase in structural definition: the system loses about 25% of the order that was originally gained in the first part, while complexity is still slightly increasing. In the final stretch for t > 8000, another minor increase in order is observed, while complexity levels out to a constant value. 

Despite the partial loss after 2000 time steps, a clear development towards increased order and complexity along the diagonal is observed. This, again, is a valid proof of the potential of this process to contribute to a structural evolution. Near the final point at 20,000 time steps, the complexity is determined by a superposition of the three different composomes according to Equation (4), whereas the structural order is derived from the dynamic state between composome populations *P*_1_, *P*_2_, and *P*_3_ according to Equation (2). In this state, the system already reaches a certain degree of primitive functionality, represented by the steady state equilibrium between three types of composomes and its possible reactions to the variation in external parameters. Even though it may be a tiny step on the ladder of an evolution process, it represents a shift in the adequate direction of increasing order and complexity, leading to basic functionality. The molar entropy change connected to the overall increase in order is given by ΔS = −12.14 J/(K∙mol). This value describes a real thermodynamic property of the composome formation process, an actual loss of micellar entropy upon transition from high entropy initial random micelles to the more admixture–orderly composomes. Nevertheless, composome formation—just like the general micelle formation—has a net driving force. In a real system, this negative entropy change is overcompensated by the negative free enthalpy change connected to specific interactions between the amphiphiles. The latter involves the positive entropy change in the hydrophobic interactions of the amphiphile tail–tail adherence in water as well as (in some cases) the negative enthalpy change connected to specific interactions between the amphiphiles. In the simulation, the driving force is represented by the particular setting of the β-matrix and the given relations between forward and backward rate constants.

### 5.4. Possible Micellar Evolution

Developments, as shown in Section 5.3, may lead to a dynamic set of composomes but clearly do not have the potential to reach states of higher degrees of functionality. As obvious from the plots in Figure 4 and Figure 6, the reactor reaches a steady state with respect to the ratio among different composomes. After approximately 10,000 time intervals, no further optimization occurs. The latter is expected as the reactor is at a steady state and the underlying *β*-matrix is unchanged. However, this also means that such a single development towards a steady state is quite limited in its power to push the progress along the diagonal in the order/complexity scheme and to evolve the system towards functionality. 

A real evolution process requires externally induced changes that will drive the system towards a repeated sequence of different steady states. Such repetitive changes could be initiated by variations in the chemical environment (e.g., connected to the appearance of high-energy volcanic sulfur and phosphorous compounds that could lead to novel endogenous syntheses [12]). Alternatively, they could be induced by cyclic variations in physical conditions, as given by wet/dry cycling in open ponds on a daily basis [27,28,29], or by cyclic pressure changes induced by geyser activity or tidal influences [30,31]. Any such repeated environmental changes could force a re-adjustment of the steady-state balance towards novel composomes. 

**Figure 8:**
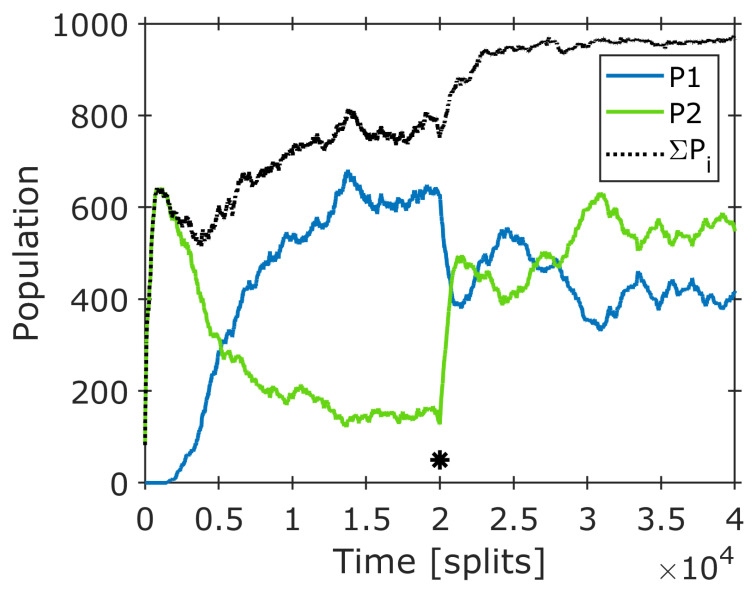
Time dependence of the propensities *P_k_* of composomes *k* within a constant population reactor under the influence of a sudden change in the individual concentrations of the amphiphiles (equivalent to a sudden change in *π_i_*). The asterisk indicates the point of the concentration change on the time axis, the black dotted line marks the sum of both composome contributions (*P*_1_ + *P*_2_) [12].

The consequences of such parameter changes (generally affecting amphiphile concentrations) on the composome formation have been demonstrated in another numeric simulation (Figure 8) [12]. In the first part (t < 20,000 steps), the plot shows the development of two composomes with *P*_1_ ≈ 0.18 (green) and *P*_2_ ≈ 0.62 (blue) in the final steady state. At t = 20,000 steps, a sudden concentration change is applied, leading the system into a new equilibrium at *P*_1_ ≈ 0.55 (green) and *P*_2_ ≈ 0.40 (blue). 

Hence, the parameter change from A to B leads the system into a new composome setting. If such a change occurs periodically on a short time scale, the system is kept in a permanent non-equilibrium, and the equilibrium is avoided. Such a concentration change is necessarily induced by periodic influences such as wet/dry cycling or pressure-related phase transitions. Under these conditions, one state (e.g., the dry one) is more prone to molecular variation, while the other (e.g., the wet one) could induce structural stress, leading to a loss of more sensitive varieties. For simplicity, the first step could be assigned to mutation, and the second would represent selection. In combination and in periodic repetition, both steps would form the key elements of Darwinian evolution. 

**Figure 9:**
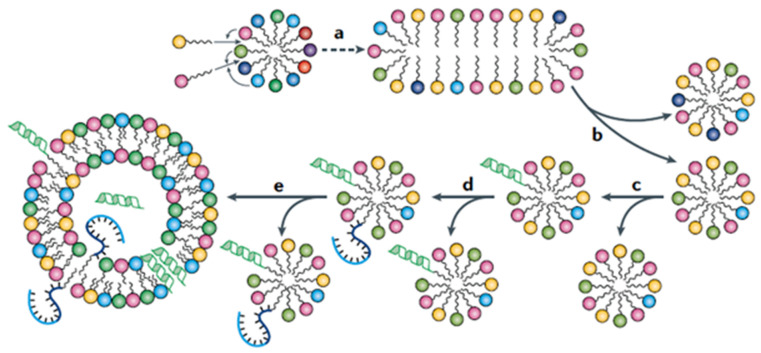
Micellar reproduction and evolution. (a) According to the graded autocatalysis replication domain model, mutually catalytic interactions between micellar lipids and free lipidic monomers in solution influence the rates of entry (and exit) of monomers into (and from) the micellar assembly. On catalyzed growth, micelles are shown to eventually assume specific lipid compositions (composomes) with narrower lipid repertoire. Graded autocatalysis replication domain simulations show that such kinetically instructed compositions exhibit homeostatic growth. (b) The grown micelle splits while transmitting its compositional information to the next generation, in other words, it is reproducing. (c,d) On growth and splitting, homeostatic compositional reproduction (curved arrows) with mutations (straight arrows) may involve both non-covalent accretion depicted in panel (a) and covalent lipid modifications, as occurring in certain catalytically reproducing heterogeneous micelles. These reactions could, among others, involve catalyzed oligomerization of micelle-attached amino acids and nucleotides (green, α-helical peptide; blue, short-folded RNA). The latter lipid endogenous syntheses obtain experimental basis from studies of the formation of nucleoside, nucleotide and oligonucleotide amphiphiles, with additional diversification towards lipid headgroup being peptide–oligonucleotide coupling products (see text). (e) Some lipid modifications, such as a conversion of single-chain to double-chain lipids, could result in a micelle-to-vesicle transition, including the addition of core content and transmembrane molecules. The sequence of events depicted here portrays a gradual progression from micelles that undergo purely compositional inheritance to more elaborate vesicular protocells that embody sequence-based replication and core metabolism. It is important to stress that the term composome applies to all the lipid entities, micellar and vesicular, as well as all their early protocellular descendants, all of which have the capacity to self-reproduce in their entirety [13] (From [13] with permission).

A schematic pathway of a micellar evolution was previously presented by Amit Kahana et al. [13]. A summary of possible processes connected to reproduction and a postulated evolution is shown in Figure 9. We note that the top row represents reproduction with small catalyzed compositional mutations affected by the influx and outflux of molecules belonging to a pure, non-covalent evolutionary arena. In contrast, in the bottom row, metabolic GARD dynamics with catalyzed covalent synthesis prevail in addition to non-covalent changes [12]. This conducts a much more significant complexification path, which is to be studied in detail in future endeavors.

**Figure 10:**
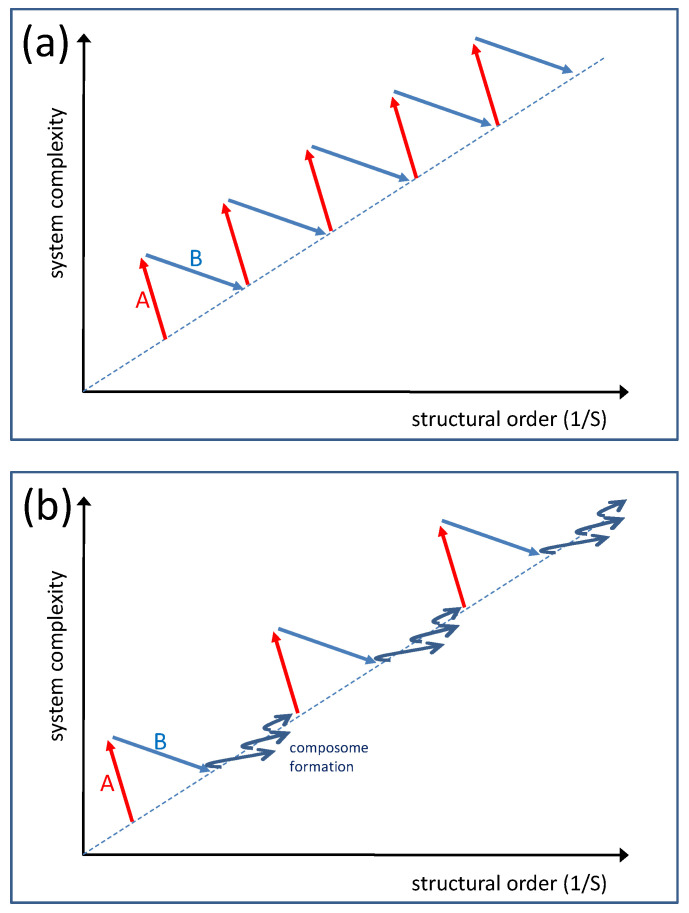
Expected qualitative order–complexity diagrams symbolizing possible pathways of evolution: (**a**) Sequence of alternating parameter settings A and B with A being associated with mutation, B being associated with selection; (**b**) sequence of alternating parameter settings A (mutation) and B (selection), including periods of composome formation similar to the one shown in Figure 5 and Figure 7.

In this scenario, the mutation steps include covalent molecular modifications and polymerizations that are more stably preserved. These changes would lead to functionalized micelles with significantly altered properties and likely advantages in natural selection processes. The power of repeated mutation and selection processes in terms of driving productive evolution is immense. The occurrence of compositional mutation, both covalent and non-covalent, incites the variability of the system and dramatically increases its complexity while a significant amount of order is lost. The subsequent selection reduces complexity since many varieties will disappear. On the other hand, it increases the order because the selectivity of the environment always favors a certain structural feature that will accumulate, leading to more defined structures. At every starting point of a new cycle, there is a more complex and ordered situation compared to the initial position of the cycle before. Therefore, in an order–complexity diagram, the overall development follows a characteristic zigzag course that is typical of an evolution process (Figure 10a) [19,20]. 

This zigzag process superimposes the formation of new, more complex, and ordered composomes during each step (e.g., as shown in Figure 5 and Figure 7). In a simplified representation (with separated mutation, selection, and composome formation steps), the overall course may look like that in Figure 10b. 

Altogether, the micellar and later vesicular systems continuously increase the order and complexity and may achieve new functions. It will consist of numerous composome populations, some specialized for setting A and some for setting B. These different composome populations may compete or cooperate and altogether form something like a complex ecosystem [26]. At one point, micelles will likely undergo significant morphological transformations, e.g., accumulate compositional mutations leading to micelle-to-vesicle transformation based on lipid geometry variations (Figure 9e). Related experiment-based structural changes in the lipid world have been reported [32,33,34,35,36,37], including oligo-nucleotide-based amphiphiles and experimental attempts to obtain morphological changes in lipid structures through biomolecular interactions under prebiotic conditions [38,39,40,41,42]. As micelles can join vesicles by membrane fusion, their evolution can be fed into larger and more complex vesicular or even protocellular structures and enrich them with endogenous catalytic synthesis and membrane transport capacities [33,35]. These functionalities will ensure that a given degree of order will be provided to the evolving entities along with the given degree of complexity. Without this functionality, the order of the system would soon be lost in spontaneous developments following the second law of thermodynamics. Hence, the functionality of the system survives in a constant battle against the natural driving force of increasing entropy. 

When compared to the molecular evolution in an RNA world scenario [20], significant differences are obvious. The RNA development does not have a substantial limitation in terms of order and complexity. RNA chains may achieve any degree of complexity by becoming longer and longer and, at the same time, achieving almost any degree of order by increasing the definition of their sequence. In contrast, the evolution of a micellar system is limited in terms of both order and complexity. The reliability in terms of micellar reproduction (Figure 9a,b) will not reach the quality of a nucleic acid chain replication, even for ideal composomes. This results in a serious limitation regarding order. In addition, the size of the micelles (in terms of the number of integrated amphiphilic molecules) will not exceed a certain structural limit. Even though many composomes may coexist, this results in a corresponding limitation regarding complexity. 

Nevertheless, the potential of GARD-driven lipid micelles for Darwinian evolution is striking. On the one hand, simple as they are, micellar composomes can self-reproduce and bequeath their chemical composition to progeny, including their mutually catalytic networks. In parallel, as described above, micelles provide the basis for the formation of much more complex and ordered lipid protocellular structures, along with possibly founding micellar ecosystems of considerable structural order and complexity. Resulting from their simplicity and straightforward continuity towards significantly evolved entities, micelles may serve as a solid basis for crucial steps in the origin of life. All this given, it would be highly interesting to empirically study micelle self-replication [38,39], as also reviewed in [12,13].

## Data Availability

No relevant research data.

## Supplementary Materials

The following are available online at https://www.mdpi.com/article/10.3390/life14030288/s1, Supporting Information: Justification of Equation (2).
