# Supplementary material for: The GARD Prebiotic Reproduction Model Described in Order and Complexity
Source: Life (Basel). 2024 Feb 21;14(3):288. doi: 10.3390/life14030288 (PMC10971654; doi:10.3390/life14030288)
Supplement: Supplementary file 1 [file life-14-00288-s001.zip › life-2872091-supplementary.pdf]

Supporting Information: Justification of Eq. [2]

A) General approach for a sequential entropy

In statistical thermodynamics, the term entropy is defined according to the Boltzmann formula

$$S = k_B \ln w \quad [S1]$$

with k_B as Boltzmann's constant and w as the statistical weight of the system's state. We assume that a system consists of N separate amphiphilic units of different varieties $i = 1, 2, 3, \ldots n$. If each variety i occurs at a number of m_i (with $m_1 + m_2 + m_3 + \ldots + m_n = N$), the number of different sequences obtained in a random walk through all of the units (and hence the corresponding statistical weight of the system's state) is given by

$$w = \frac{N!}{m_1! \times m_2! \times m_3! \times \ldots . \times m_n!} \quad [S2]$$

Or, in a logarithmic form:

$$\ln w = \ln N! - \ln m_1! - \ln m_2! - \ln m_3! - \ldots - \ln m_n! \quad [S3]$$

With the approximation $\ln x! \approx x \ln x - x$ for large numbers x and accounting for $m_1 + m_2 + m_3 + \ldots + m_n = N$, we get

$$\begin{aligned} \ln w &= N \ln N - \sum_{i=1}^{n} m_i \ln m_i \\ &= \sum_{i=1}^{n} (m_i \ln N - m_i \ln m_i) \end{aligned} \quad [S4]$$

When we introduce the fraction $p_i = m_i / N$ of each variety i, this expression turns into

$$\begin{aligned} \ln w &= -N \sum_{i=1}^{n} p_i \ln p_i \\ &= N \sum_{i=1}^{n} p_i \ln (1/p_i) \end{aligned} \quad [S5]$$

Together with the Boltzmann formula Eq. [S1], this leads to a term for the sequential entropy:

$$S = k_B N \sum_{i=1}^{n} p_i \ln (1/p_i) \quad [S6]$$

In the case of dissolved amphiphiles, the micellar populations p_i are replaced by the solution populations π_i. The result shown in Eq. [S6] is completely analogous to the thermodynamic entropy of N particles with n different states $i = 1, 2, 3, \ldots n$ and given populations p_i where $p_1 + p_2 + p_3 + \ldots + p_n = 1$.

In a system that consists of only one single type of unit (that is, $p_1 = 1$ while all other populations are zero), all random walks would yield identical sequences. According to Eq. [S6], this leads to $S = 0$. In a system that consists of, e.g., 30 different units with equal populations $p_i = 1/30$, the corresponding result is $S = \mathrm{k}N \ln 30$.

<u>B) A characteristic random walk</u>

We now need to consider an averaged characteristic random walk following the rules that are described in section 3. Assuming that the system consists of amphiphilic molecules in solution as well as of micelles that contain an average number of N amphiphilic molecules, we assume the random walk will connect an average of M free amphiphilic molecules before it hits the first micelle. Starting from this point, the path connects all units of the micelle following the rule that it always continues to the next neighbor that has not yet been part of the random walk. When all steps through the micelle are finished, the random walk again continues in the solution, starting with the next neighbor. Thus, on average, a typical step, including one path through the solution and one path through a micelle (or the other way round), includes $M + N$ units. Considering a random walk over a total of N_{rw} units, we need to account for $N_{rw}/(M + N)$ repetitions of this step.

During each such step (one path through the solution and one micelle), two different contributions to the sequential entropy have to be accounted for: i) the path through the monomers in the solution, and ii) the path along the units of the micelle. Both contributions are considered in the following.

1) Path through the solution of amphiphiles

This contribution to the sequential entropy is determined by M as the number of monomer units per step and by the relative populations π_i of the N_G different types of amphiphiles where $\pi_1 + \pi_2 + \pi_3 + \ldots + \pi_{NG} = 1$. In analogy to Eq. [S6] and for $N_{rw}/(M + N)$ steps, it reads

$$S_1 = \mathrm{k_B}\, M\left(\frac{N_{rw}}{M+N}\right) \sum_{i=1}^{N_G} \pi_i \ln\left(1/\pi_i\right) \qquad \text{[S7]}$$

For just one single type of amphiphile and $\pi_1 = 1$, this contribution is zero. For, e.g., 30 different types of amphiphiles with equal populations $\pi_1 = \pi_2 = \ldots = \pi_{30} = 1/30$, it turns into $(\ln 30)\, \mathrm{k_B}\, MN_{rw}/(M + N)$.

2) Path along the units of the micelle

The second contribution to the sequential entropy is determined by the relative predictability p_i of each following amphiphile molecule of the type i within a micelle. This predictability is defined by the composition of the micelle and the corresponding relative contributions of its amphiphilic molecules p_i with $p_1 + p_2 + p_3 + \ldots + p_{NG} = 1$. In analogy to Eq. [S6] and for $N_{rw}/(M + N)$ steps, the contribution S'_2 for one single type of micelle consisting of N amphiphilic molecules reads

$$S'_2 = k_B \left(\frac{N_{rw}}{M+N}\right) N \sum_{i=1}^{N_G} p_i \ln(1/p_i) \qquad [S8]$$

For a micelle that contains only one single type of amphiphile with $p_1 = 1$ (and all other $p_i = 0$), this contribution is zero. For completely random micelles of, e.g., 30 different amphiphiles and all $p_i = 1/30$, it turns into (ln 30) k_B $NN_{rw}/(M + N)$.

In general, the assembly will contain several groups of micelles that will follow different preferred compositions, some of them deviating from the composition of the solution. This will be especially the case when composomes form. These may be characterized by an index k and individual contributions $p_{i,k}$ of their amphiphiles. Considering the relative contribution P_k of micelles of the type k, the overall contribution S_2 now reads

$$S_2 = k_B \sum_k \left[\left(\frac{P_k N_{rw}}{M+N}\right) N_k \sum_{i=1}^{N_G} p_{i,k} \ln(1/p_{i,k})\right] \qquad [S9]$$

In combination, both contributions S_1 and S_2 add up to Eq. [2] in the main text. If N_{rw} is set to $6.022 \cdot 10^{23}$, the resulting value $S_r = S_1 + S_2$ accounts for a molar entropy of the assembly with respect to its amphiphilic composition. Its thermodynamic equivalent is the mixing entropy. This mixing entropy would contribute to the driving force for the complete loss of the composomal structure.
